# Sound Asleep: Processing and Retention of Slow Oscillation Phase-Targeted Stimuli

**DOI:** 10.1371/journal.pone.0101567

**Published:** 2014-07-07

**Authors:** Roy Cox, Ilia Korjoukov, Marieke de Boer, Lucia M. Talamini

**Affiliations:** 1 Department of Psychology, University of Amsterdam, Amsterdam, the Netherlands; 2 Amsterdam Brain and Cognition, University of Amsterdam, Amsterdam, the Netherlands; 3 Okazolab Ltd, London, United Kingdom; University of Oxford, United Kingdom

## Abstract

The sleeping brain retains some residual information processing capacity. Although direct evidence is scarce, a substantial literature suggests the phase of slow oscillations during deep sleep to be an important determinant for stimulus processing. Here, we introduce an algorithm for predicting slow oscillations in real-time. Using this approach to present stimuli directed at both oscillatory up and down states, we show neural stimulus processing depends importantly on the slow oscillation phase. During ensuing wakefulness, however, we did not observe differential brain or behavioral responses to these stimulus categories, suggesting no enduring memories were formed. We speculate that while simpler forms of learning may occur during sleep, neocortically based memories are not readily established during deep sleep.

## Introduction

Sleeping organisms show a much-reduced responsiveness to external stimuli, indicating a relative disconnection of their brains from the environment. While this daily recurring suspension of ‘online’ processing comes at a risk, it also allows brains to perform 'offline' tasks they cannot carry out during the constant sensory bombardment of wakefulness. In particular, the last decades have demonstrated the various effects that sleep has on memory stabilization and reorganization [1], [2], processes that depend on the replay of previously learned information [3], the temporal coupling of various brain rhythms [4], and the gradual redistribution of memory traces from temporary to more permanent stores [5]. Such findings have instilled the realization that important information processing may be ongoing during sleep. Yet, how the sleeping brain reprocesses stored information is an issue largely distinct from whether and how the sleeping brain processes incoming stimuli. Particularly notable in this respect are some recent reports indicating the retention of information presented during sleep, that is, sleep-learning [6], [7]. However, stimulus processing during sleep has been examined from a number of research angles.

One recent line of evidence for the sleeping brain's lingering receptiveness to stimuli comes from studies using external cues to reactivate memory traces [8], [9], leading, in some cases, to improved memory performance [10], [11]. Other studies have evaluated sleeping subjects' brain responses to novel input, usually simple auditory or tactile stimuli. A number of human studies investigated evoked potentials in response to stimulus presentation during sleep (for a review see [12]). The general tenet from this body of work is that, while the sleeping brain responds differently from wakefulness, it retains some residual capacity for performing simple processing relating to stimulus salience, novelty and significance, as indicated by differential brain responses to stimuli varying in these respects. At the same time, such electrophysiological responses depend on the particular sleep stage the subject is in, and at a more fine-grained level, on the momentary presence of specific brain oscillations [13]–[16].

In particular, the phase of ∼1 Hz slow oscillations (SOs) during deep, or slow wave sleep (SWS), appears to affect stimulus processing. During SOs, membrane potentials of neocortical circuits alternate between depolarized up and hyperpolarized down states. In line with the notion that most faster activity occurs in the up state [17]–[19], and the brain is generally more excitable during this SO phase [20], a recent study found evidence that auditory stimuli arriving during the upward going SO slope (i.e., towards the up state) lead to greater hemodynamic responses in auditory association cortex, as compared to tones presented during the negative-going wave (i.e., towards the down state) [16]. In the same study, tones presented towards the up state elicited a greater evoked potential positivity ∼300 ms post-stimulus than tones presented towards the down state. In contrast, somatosensory evoked potentials to brief tactile stimuli were largest in the down state and became progressively smaller towards the up state [15], complicating the question as to which phase of the SO is most conducive to stimulus processing.

As alluded to above, perhaps most intriguing is whether novel information, processed during sleep, can leave lasting memory traces discernible during ensuing wakefulness. Older studies found either no evidence for sleep-learning of verbal material [21], [22] or had methodological difficulties [23]. However, recent evidence demonstrated that classical conditioning can take place during sleep [6], as can fear extinction learning [7]. A possible explanation for these inconsistencies may have to do with the neural networks likely involved in forming memories for the presented materials. While the successful conditioning and extinction attempts probably relied on subcortical and hippocampal brain circuitry, the failure to establish memories for verbal material may be related to their dependence on neocortex. In particular, the precise timing of stimulus delivery may be of crucial importance. If indeed SOs, with their neocortical basis, regulate windows of opportunity for stimulus processing, it may be that stimuli processed in large part by the neocortex need to arrive sufficiently often in the right SO phase in order to be encoded properly and leave a permanent mark on brain circuitry.

Thus, if one were to assess the role of SOs in auditory stimulus processing and memory formation, complex, real-world sounds with semantic meaning ('sound objects') may be preferred over simple tones. Importantly, the buildup of lasting memory representations is a function of the number of times stimuli have been encountered. As such, the ability to present stimuli repeatedly and consistently in a specific SO phase would be highly useful and maximize the opportunity to demonstrate SO phase-dependent learning. A related benefit of such a capability would be that meaningful sound stimuli, which are usually of longer duration, will not end up along different SO phases, thereby preventing a straightforward examination of up and down state-related processing.

In this study, we examined how cortical networks respond to real-world sound stimuli as a function of SO phase. In order to achieve this, we developed a novel algorithm for performing real-time phase prediction in the SO frequency range. Using this algorithm, we repeatedly presented sound stimuli directed at up and down states to sleeping subjects. Importantly, by consistently targeting particular stimuli at a specific SO phase, we putatively engendered the gradual buildup of a memory trace. We evaluated brain-wide SO phase-dependent brain responses to stimuli in both the time and time-frequency domain. We analyzed time-frequency power responses because time-varying spectral responses may be affected even when time-averaged signals are not [24]. Finally, to assess whether lasting memory traces were formed, we administered all presented, as well as novel, sounds during a post-sleep waking session, and explored neural and behavioral indices of memory formation.

## Materials and Methods

### Ethics statement

This study was conducted in accordance with the principles of the Declaration of Helsinki, procedures were approved by the University of Amsterdam, Department of Psychology ethics committee, and all participants provided written informed consent.

### Subjects

A total of 12 young, healthy participants (age range 18–23; 11 female), for whom algorithm-based stimulus delivery was successful (see Results), were included in this study. Subjects were compensated either monetarily or with credits to fulfill course requirements.

### Procedure

Participants arrived at the sleep laboratory at 8 PM. After filling out of informed consent forms, subjects were prepared for polysomnographic registration (see Data acquisition). Participants went to bed between 9 PM and 9.30 PM, when they were provided with a 2 to 2.5 h sleep opportunity in a dark and quiet environment. Speakers placed approximately 50 cm from the subject's head played white noise continuously at an unobtrusive level; subjects had been informed the goal of the experiment was to learn about sound processing during sleep. The real-time polysomnographical signals were constantly monitored by the experimenter from the control room adjacent to the subject's bedroom. The experimenter turned the phase prediction algorithm on and off to coincide with the presence of SWS. When several criteria were met (see Algorithm), the algorithm triggered playback of a sound stimulus, while temporarily pausing the white noise. Out of a total of 60 sounds, 20 were directed at the SO up state, and 20 at the down state. The remaining 20 stimuli were kept separate for later testing during wakefulness. The algorithm was allowed to predict and play sounds for as long as the participant remained in SWS. Some subjects woke up after an initial period of successful stimulus presentation and did not show signs of falling asleep again. In that case, they were disconnected from the polysomnography setup after lying awake for 45 min. Subjects who slept steadily were woken up from light or REM sleep after 2 to 2.5 h.

After a period of 30 min to recover from sleep inertia, subjects were asked to fill out the Stanford Sleepiness Scale and answer questions regarding general sleep characteristics (not pertaining to the nap). In addition, they were asked whether they had noticed anything out of the ordinary during sleep, and, more specifically, whether they had noticed anything about the background noise. Subjects were then informed that sounds other than the white noise had in fact been played. After rechecking electrode impedance levels, participants performed a waking memory test with EEG registration (see Memory task), during which sounds from the three conditions (up/down/novel) were presented in pairs. Subjects were required to indicate which sound of each pair sounded most familiar, using a forced choice design. In this way, we could assess whether memories had been formed during sleep that could guide behavioral choices, either consciously or subconsciously. In particular, one might expect that up and down sounds will be consistently chosen over novel sounds, indicating 'generic' sleep-learning. Alternatively, up sounds might be chosen over both down and novel sounds, suggesting sleep-learning occurs particularly in the SO up state. Lastly, an exit questionnaire assessed subjects' confidence regarding their provided answers. Total duration of experimental procedures was between 3.5 and 4.5 hours.

### Stimuli

We selected a total of 60 sound stimuli representing real-world objects (door bell, barking dog, footsteps, etc.). Some of these stimuli have been used previously [8], [11], others were found through the Internet. The maximum duration of the sounds was 500 ms, so that they would 'fit' into individual up or down states. Our goal was to have the middle of each sound clip coincide with the SO peak or trough. In order to deal with different sound durations, for sleep presentation, sound files were edited to be 500 ms in length by zero-padding them symmetrically to the required length. Thus, the effective stimulus was always centered in the middle of the 500 ms sound clip. We also employed a white noise stimulus, which, during playback, was constantly looped. Empty padding was not performed for stimuli presented during wakefulness testing. Sound levels of all sounds, including the white noise, were equalized using the 'account for perceived loudness' option in Adobe Soundbooth. All sounds were stereo waveform files with a sample rate of 44.1 kHz. Sound levels during sleep were set at an unobtrusive volume, ranging from 35 to 45 dBA for individual sounds, which was sufficiently soft to not awake the participant but still be perceptible. During wake testing, sound volume ranged between 60 and 80 dBA.

### Data acquisition

EEG was acquired using a 64-channel WaveGuard cap (ANT, Enschede, the Netherlands) and two additional fixed mastoid electrodes. Horizontal and vertical electrooculography and chin electromyography were monitored with bipolar derivations. Signals were sampled at 512 Hz using a 72-channel Refa DC amplifier with a low-pass filter at one fifth of the sample rate (TMS International, Enschede, the Netherlands), and stored on a recording pc. EEG was acquired with an average reference and impedance levels were kept below 10 kΩ. During sleep, a second amplifier provided the bipolar Fpz-M1 signal to the algorithm pc, which carried out phase prediction.

### Algorithm

In order to present stimuli in a specific phase of the SO in real-time, we developed a novel algorithm. The algorithm runs in the Event IDE (http://okazolab.com) environment on a dedicated pc, receiving the signal of a selected EEG channel (bipolar Fpz-M1), through a dedicated amplifier. The last 5,000 samples (∼10 s) were buffered and used for analysis. In a continuous fashion, a Fast Fourier Transform (FFT) was applied to the moving window of these most recent samples to analyze the spectral composition of the signal. We designated the frequency with the highest amplitude coefficient in the 0.6–1.2 Hz range as the center frequency, serving as an estimate of the momentary SO frequency. Next, we band-pass filtered the signal in the SO band (order 1 Butterworth, 1 Hz bandwidth around center frequency). Then, using the Hilbert transform, the analytic signal was derived. Because the analytic signal is phase-shifted 90 degrees with respect to the original signal, it was shifted back. An important feature of the analytic signal is that its cycle-to-cycle maximum amplitude is constant; amplitude differences from one SO to the next are thus eliminated. Using the previously determined center frequency, a sine wave was fitted to the analytic signal and extrapolated into the future. Importantly, for fitting we only used the period between 80 and 95% (∼1.5 s) of the analytic signal. We used this smaller piece of data to use only the most recent bit of data for extrapolation. However, because of edge artifacts when filtering, we were forced to stay clear of the most recent ∼0.5 s. For a given target phase, the first occurrence of that phase along the extrapolated sine wave was used to estimate its expected occurrence.

Without any further requirements, however, this would lead to a near-continuous stream of predictions, since there was always an extrapolated signal. Therefore, we enforced a number of additional criteria. First, in order to minimize predictions in the absence of SOs, we set a power threshold that power in the SO band was required to exceed. Specifically, power in the SO band should be at least a factor 0.6 of the total power. While this does not guarantee the presence of deep sleep (e.g., eye movements at the right periodicity could also result in crossing the power threshold), this approach severely reduced the occurrence of phase predictions in non-desired vigilance states. Second, we set a fitting threshold parameter, which determined how good the fit between the sine wave and the analytic signal should minimally be. This fit was calculated using a least squares approach, and was required to be smaller than 0.1. As soon as power and fitting criteria were met, the algorithm was allowed to predict the next occurrence of the targeted phase. It would then temporarily halt online analyses and wait for the predicted time point before sending a trigger to the stimulus pc, leading to stimulus presentation. However, this momentary lapse in signal fitting meant that the most recent information was not utilized, possibly leading to less accurate performance. Therefore, as a third criterion, we set a time constraint such that predicted phases could not be more than 250 ms away from the present moment. In this manner we balanced the need for a high prediction rate, with the need for accurate predictions based on the most current information. In rare cases, sound presentation led to arousals or waking up. Therefore, after each prediction and ensuing stimulus presentation, we enforced a 10,000-sample (∼20 s) 'time-out' period, during which no predictions were made, allowing the operator to manually disable algorithm functioning if necessary. Given that there were two phases of interest (up and down state), the algorithm was continuously looking for both. Whichever phase was predicted to occur first, was the one used for stimulus presentation.

Stimuli were presented by a dedicated stimulus pc, also using Event IDE as the presentation platform. Per subject, stimuli were randomly assigned to one of three categories (up/down/novel). Sounds assigned to the up and down conditions were presented in random order. Constant white noise was played at low volume while the input port was monitored for pulses from the algorithm pc indicating the prediction of an up or down state. Upon pulse detection, noise playback was muted and the sound next in line (from the appropriate condition) was presented. White noise was then restored to the normal volume. Note that up- and down-targeted sound presentation was intermixed, as it depended on the algorithm's predictions. Following the presentation of a full block of 20 presentations from a condition, sounds in that condition were reshuffled for the next presentation block. The operator had the option to adjust sound levels in case sound presentation led to arousals or signs of waking up. For two subjects this was the case, and volume levels were lowered by about 5 dBA.

Note that, contrary to another recently described 'closed-loop' algorithm for delivering sounds in phase with SOs [25], our algorithm is capable of targeting any desired phase. Indeed, we made full use of this capacity not only because some stimuli were directed at the up state and others at the down state, but also because we took into account the dominant SO frequency when computing at what exact phase sound playback should commence. That is, a slower SO frequency meant that stimulus presentation should start slightly later and in a different phase compared with a quicker SO frequency.

### Memory task

Right before wake testing, subjects were informed that sounds had been played during their nap. They were then seated approximately 50 cm in front of a pc screen and speakers, for a memory task with EEG recording. Subjects were required to perform a 2-alternative forced choice task as to which sound of a pair appeared most familiar to them. Participants were requested to sit still and look directly at the screen in front of them. They were presented a sequence of sound pairs, in which each pair consisted of sounds from two distinct categories (up/down/novel). Every 'pair condition' (up/down; up/novel; down/novel) occurred equally often. Each of the 60 sounds was presented twice, both times in a different pairing. That is, 30 pairs were presented in round 1, and 30 different pairs (but constructed from the same sounds) in round 2. Furthermore, every sound was paired with sounds of both remaining categories over the two rounds. That is, an 'up' sound that was matched with a 'down' sound in round 1, was paired with a 'novel' sound in round 2. Finally, the order of presentation of pairs within each round was randomized, as was the order of sounds within each pair.

After presentation of a few instruction screens, sound pairs were presented. A single trial commenced with a blank-screen pre-stimulus period jittered between 1000 and 1100 ms, before the first sound of a pair was presented. Then, there was a silence (jittered between 5000 and 5100 ms), before the second sound was presented. A 4000 ms pause ensued, followed by the appearance of a question on the screen: 'which sound appears more familiar to you?' Subjects could indicate their choice by selecting one of two buttons on the keyboard. Then, a message 'the next two sounds will be presented shortly' was displayed for 2000 ms, and the next trial commenced. Following presentation of the first 30 pairs, a short 30 s break was provided. The waking test took about 15 min.

### Preprocessing

Sleep stages were scored offline using Galaxy software (PHIi, Amsterdam, the Netherlands) with an epoch size of 30 s in accordance with standard criteria [26]. In addition, periods of arousal were marked in accordance with the AASM guidelines [27]. Stimuli presented within a 2 s range around these intervals were excluded from the analyses, both for sleep and waking EEG data. Custom Matlab scripts were combined with several freely available toolboxes for all subsequent analyses. Functions from the EEGLAB toolbox (http://sccn.ucsd.edu/eeglab) were used to high-pass (0.1 Hz) and notch filter (50 Hz) the raw EEG data, interpolate channels displaying artifacts, and re-reference the EEG to linked mastoids.

Next, eye blinks, eye movements and muscle artifacts were removed from the waking EEG data using independent component analysis. For both sleep and wakefulness, continuous EEG was initially epoched into intervals from 1000 ms before until 2500 ms after sound onset. Trials were visually inspected for artifacts and noisy trials were discarded. For ERP analysis, data was then low-pass filtered at 35 Hz; for time-frequency analysis this step was not performed. All data were then baseline-corrected using an interval from −750 to 0 ms. For time-frequency analysis, a family of complex Morlet wavelets was used to decompose all multi-channel epoched time series into time-frequency representations, according to *e^i^* ^2*πtf*^ *e* ^(−*t*∧2)/(2*σ*∧2)^, where *i* is the imaginary operator, *t* is time, *f* is frequency (35 logarithmically spaced frequencies between 5 and 100 Hz), and *σ* is the width of each wavelet. We defined width *σ* as *λ*/(2*πf*), where *λ* is the number of wavelet cycles, increasing from 4 to 12 in 35 logarithmically spaced steps corresponding to the number of frequency bins. These settings resulted in a temporal precision (*2σ*) of 255 ms at 5 Hz and 38 ms at 100 Hz, and a spectral precision [1/(*πσ*)] of 2.5 Hz at 5 Hz and 16.7 Hz at 100 Hz. The resulting time-frequency representations were downsampled to 100 Hz to reduce the amount of data. Power was defined as the squared complex magnitude of the convolution result, while phase was defined as the angle of the convolution result, bound between -pi and pi. Power estimates were decibel normalized according to dB power  =  10*log10(power/baseline), where for each channel and frequency, the baseline was the average from -200 to 0 ms over all epochs. For sleep, we used separate baselines for the up- and down-targeted conditions, because of widely different pre-stimulus amplitudes and power levels in various frequency bands between up and down states. For wakefulness, pre-stimulus amplitudes and power could be assumed to be highly similar across conditions and we used the same baseline for all conditions (namely, the average baseline across all trials in all three conditions).

### Statistics

Algorithm performance was assessed offline by first processing the channel used for predictions in a manner closely resembling the processing steps employed by the real-time algorithm. That is, we band-pass filtered channel Fpz (0.5–1.5 Hz, zero-phase shift Butterworth) and used the Hilbert transform to extract the instantaneous phase. We used functions from the CircStat toolbox for circular statistics [28] to analyze the phase distributions of presented sounds. Permutation-based statistical analyses on time-channel data (for ERPs) and on time-frequency-channel data (for time-frequency power responses) were performed with Fieldtrip (http://fieldtrip.fcdonders.nl/) using cluster correction [29]. EEG statistics were performed on an interval from −200 ms to 1500 ms around stimulus presentation. We set the clusteralpha parameter to 0.01 and used 1000 iterations for all tests. Using a significance level of 0.05, clusters were considered significant at P<0.025 for two-sided testing.

## Results

### Sleep characteristics

Subjects spent an average 135±11 min (mean ± SD) in bed, of which they slept a total of 103±23 min. Proportions spent in individual sleep stages were, for S1: 17±9%; S2: 44±9%; S3: 10±4%; S4: 27±12%; only two subjects showed signs of REM sleep (7 and 14 min, respectively). Total time spent in SWS (S3 + S4), during which sound presentation was intended to occur, was 37±12 min (range: 20–61). Indeed, algorithm predictions took place overwhelmingly during deep sleep (93±6%), with only a minority occurring during S2 sleep (6±6%), and negligible amounts during wakefulness and S1 sleep (0.5 and 0.2%, respectively). Number of arousals was 21.4±17.0 (range: 4–66), although only four of these were associated with sound presentation (four subjects with one arousal each). The pertaining sounds were excluded from all EEG and behavioral analyses. Following sleep, subjects answered questions as to whether they had noticed anything about the background sound. None of the participants had noticed that the continuous noise had been intermittently replaced with other sound stimuli.

### Sleep: algorithm performance

The algorithm was allowed to predict as many up and down states as possible while deep sleep continued. The mean number of up state predictions and ensuing sound presentations was 63.3±24.3, while for the down state there were 59.8±23.2 presentations. These amounts did not differ significantly [paired t test: t(11)  = 0.98; P = 0.35], indicating the algorithm was not biased. Given that a full round of presentations consisted of 20 stimuli per condition, each sound was played three times on average.

**Figure 1:**
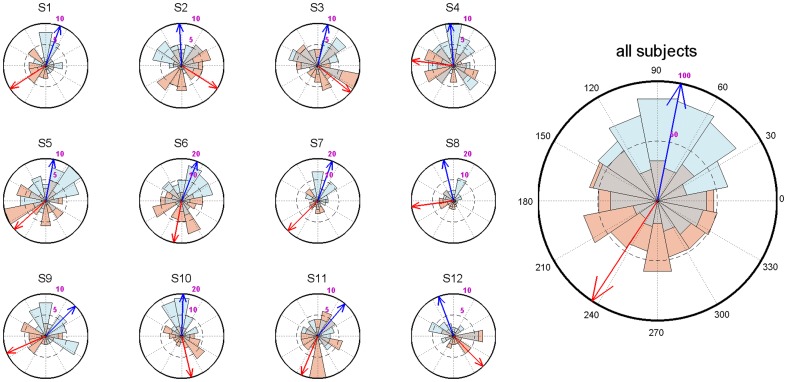
Circular histograms showing algorithm performance for up- (blue) and down-targeted (red) sound stimuli, both within single subjects and across all trials (right). Arrows indicate average phase angle; 90 degrees corresponds to the peak of the up state, 270 degrees to the trough of the down state.

For a given participant, algorithm performance needed to be sufficiently accurate before we could include that subject and sensibly compare up- with down-targeted sounds. Therefore, we analyzed the phase distributions of markers designating the middle of the sound stimuli with respect to the SO phase. In particular, we assessed whether distributions were significantly non-uniform using the Rayleigh test, and whether up- and down-targeted distributions were significantly different with the Watson-Williams test. Overseeing all subjects' algorithm performance, we included participants when 1) the average up-targeted phase deviation from the up state (90 degrees) was <45 degrees; 2) the up-targeted distribution was significantly (P<0.05) non-uniform; and 3) up- and down-targeted distributions differed significantly (P<0.05). Note that we did not explicitly require down-targeted stimuli to be <45 degrees from the down state (270 degrees), or significantly non-uniform. While for many subjects these conditions were still met, down state prediction was evidently more difficult for the algorithm than up state prediction. Nonetheless, we reasoned that with the above three requirements in place, up- and down-targeted distributions should still be sufficiently distinct to demonstrate differential brain processing. Indeed, adding all predictions from all twelve included participants together, both up- and down-targeted stimuli were presented very close to the intended phase (up-targeted: 78.7±65.6 degrees, Rayleigh test Z = 90.5, P<10^−40^; down-targeted: 236.8±75.8 degrees, Rayleigh test Z = 11.5, P<10^−4^; Watson-Williams test F = 395.2, P≈0). Figure 1 shows algorithm performance both for individual subjects and across subjects.

### Sleep: up- and down-targeted responses

**Figure 2:**
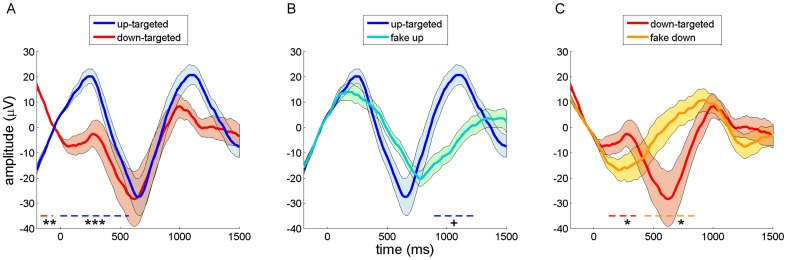
Grand-average event-related potentials during sleep for frontal channel Fz. Error shading indicates standard error of the mean. Dashed colored lines near bottom signify time period of significant difference at cluster-level, with color indicating the more positive waveform (+ P<0.05; * P<0.025; ** P<0.01; *** P<0.001). (A) Up-targeted (blue) and down-targeted (red) waveforms. (B) Up-targeted (blue) and fake-up events (turquoise). (C) Down-targeted (red) and fake-down events (gold).

In a first step, we compared ERP and time-frequency power responses between the up- and down-targeted stimuli across all electrodes. Note that these contrasts reflect putative stimulus-evoked responses against a backdrop of differential SO phases in the two conditions. For these and subsequent analyses, we time-locked all responses to actual sound onset, stripped of empty sound padding. Evoked potentials demonstrated up-targeted sounds were presented on an upward going slope, while down-targeted sounds occurred against a negative-going wave (Figure 2A). This is consistent with the differential SO phase targeting, since the contribution of SOs to ERPs would be expected to produce inverted waveforms in the two conditions. Consequently, up-targeted ERPs were significantly more positive than down-targeted ones from −40 to 580 ms (P<0.001), and more negative in the pre-stimulus period (-200 to −60 ms, P = 0.008). These effects occurred in clusters encompassing all channels, although they were most pronounced in frontal regions, closer to the site that phase prediction was based on.

Considering the entire segment, the up-targeted ERP showed a regular periodic wave in the SO frequency range. The down-targeted waveform, on the other hand, displayed a positive deflection around 350 ms, which seemed to interrupt the downward going wave and to delay its negative peak. Interestingly, the waveforms in the two conditions more or less realigned in the second part of the segment, from ∼600 ms onwards, suggesting that the sound presentations consistently altered the time course of the slow oscillation dynamic, in one or both conditions.

**Figure 3:**
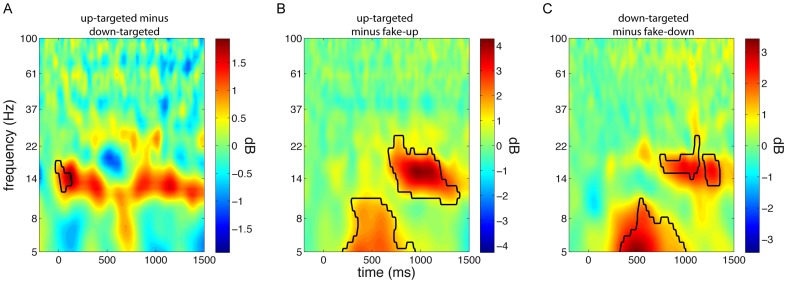
Time-frequency power difference plots for frontal electrode Fz. Conceptually, panels A-C are similar to Figure 2 A-C. (A) Up-targeted minus down-targeted sound presentation reveals an early enhancement in spindle power in the up state. (B) Up-targeted stimulus delivery compared with fake-up events results in an initial theta response, followed by a later spindle/beta response. (C) Similarly, down-targeted sounds elicit theta and spindle/beta activity relative to fake-down events.

Evoked power responses demonstrated a borderline significant increase in the spindle frequency range (10–19 Hz) for up-targeted stimuli, relative to down-targeted ones, in a window from -50 to 480 ms (P = 0.039, Figure 3A). This effect could again be observed across most of the scalp (69% of channels), but was most pronounced over frontocentral areas. This observation is consistent with findings of enhanced fast spindle activity in the SO up state [30]. Together, ERP and time-frequency data provide additional confirmation that our algorithm succeeded in presenting stimuli in the desired phase. Moreover, they suggest stimulus presentation altered SO dynamics. This notion is further explored in the next section.

### Sleep: stimulus-evoked responses

In order to demonstrate stimulus-related activity unrelated to spontaneous SOs, we defined fake events with the same SO phase distribution as presented stimuli, without actual stimulus presentation. That is, for every presented stimulus, we searched the band-pass filtered signal for the same phase in the 3 s preceding that stimulus, and labeled the corresponding time point as a fake event. We did this for up- and down-targeted stimuli separately, creating fake-up and fake-down events. Note that all fake events occurred during SWS and during ongoing SOs. By comparing up-targeted trials with corresponding fake-up trials, and, analogously, by comparing down-targeted trials with their corresponding fake-down trials, we could isolate stimulus-evoked activity from spontaneous SO-related brain dynamics.

Looking at ERPs, up-targeted and corresponding fake-up events were highly similar during the baseline period and immediately after, suggesting we were successful in selecting comparable waves (Figure 2B). However, relative to fake-up events, sound presentation led to a slightly enhanced positivity around 300 ms post-stimulus, a more negative and somewhat phase-advanced wave around 600 ms, and a more pronounced positive wave thereafter (860 to 1230 ms, P = 0.028). The cluster showing the latter effect had a frontocentral distribution encompassing 50% of electrodes. This pattern is consistent with the literature on vigorous, stimulus-evoked K-complexes [31]. Additionally, the phase-advancing effect suggests up-targeted stimuli may be able to increase the dominant SO frequency.

Down-targeted stimuli and fake events at corresponding phases also showed high similarity in the baseline period, again suggesting these waves were physiologically comparable (Figure 2C). However, compared to fake-down events, sound stimulation led to an initial positivity from 90 to 400 ms post-stimulus (P = 0.022, 81% of channels), followed by a pronounced negative peak (390–860 ms, P = 0.012, 94% of channels), possibly also indicating a stimulus-induced K-complex. This stimulus-related negative peak and the ensuing positive wave were phase-delayed compared to the baseline SO, which proceeded smoothly from down state to up state.

The spectral responses to sound events relative to fake events were highly similar for up-targeted and down-targeted stimuli (Figure 3BC). Specifically, an early theta response was maximally apparent between 200 and 800 ms post-stimulus (up-targeted: 5–11 Hz, 140–950 ms; down-targeted: 5–10 Hz, 180–1170 ms; both P<0.001), while a later broadband response, covering the spindle and gamma range, occurred from around 700 to 1500 ms post-stimulus (up-targeted: 9–35 Hz, 620–1500 ms; down-targeted: 11–29 Hz, 710–1500 ms; both P<0.001). All these effects were widespread, involving between 90 and 100% of electrodes. Timing-wise, the theta responses occurred just prior to the ERP negative peaks, at 600–650 ms, that were shared by the up- and down-targeted conditions. In contrast, the later high frequency responses aligned well with the ensuing, equally shared, positive ERP peaks.

Taken together, these findings suggest that sound presentation led to reliable brain responses. Relative to spontaneously occurring SOs, both up- and down-targeted sound presentation resulted in ERPs compatible with induced K-complexes. Interestingly, these effects combined such that around 600 ms post-stimulus up- and down-targeted waveforms were in phase again (Figure 2A). In the power spectral domain this was matched by sound-related theta responses around the peak negativity at 600 ms and, around the time of the next positivity (∼1000 ms), by an enhancement of activity in a frequency range spanning the spindle, beta and lower gamma bands.

### Sleep: SO phase-dependent stimulus processing

Having established stimulus-evoked brain responses corrected for the activity associated with naturally occurring SOs, we directly compared these responses for up- and down-targeted stimuli. That is, we calculated [up-targeted minus fake-up] and [down-targeted minus fake-down] for every subject, and compared these up- and down-evoked responses in the time and time-frequency domains across all electrodes.

**Figure 4:**
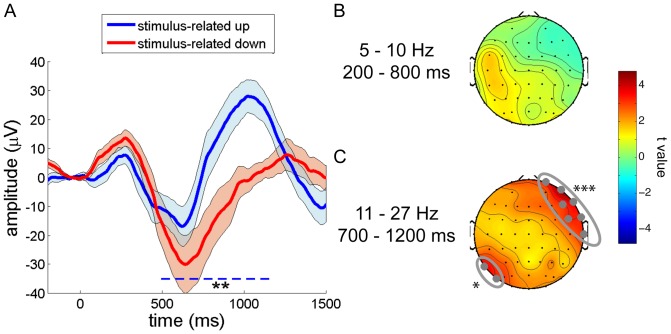
SO phase-dependent stimulus processing. Reliable differences are indicated with: * P<0.025; ** P<0.01; *** P<0.001. (A) Differential stimulus-evoked waveforms for up (blue) and down (red) state presented sound stimuli for frontal channel Fz. (B) Early stimulus-evoked theta power did not differ reliably between up- and down-targeted stimuli. (C) Late spindle/beta power was higher for up-targeted sounds than for down-targeted stimuli across the entire scalp, reaching significance in a right fronto-temporal area (electrodes Fp2, F8, FC6, T8, AF8, F6 and FT8), and a left parietal region (P7 and PO7).

ERP analyses revealed that both up- and down-targeted stimuli elicited an initial positivity around 300 ms post-stimulus, followed by a downward wave around 600 ms, and a later positivity. However, relative to down-targeted sounds, stimulus-related activity to up-targeted stimuli was reduced for the early and intermediate responses, but much enhanced for the late positive wave. This resulted in a significant difference from 460 to 1170 ms post-stimulus (P = 0.004, Figure 4A). This effect was topographically widespread, involving 76% of electrodes, although the frontal-most channels were not part of the significant cluster.

**Figure 5:**
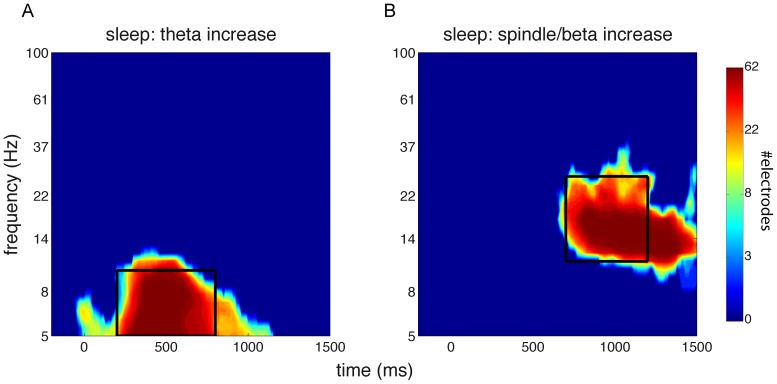
Heat maps of clusters showing significantly elevated power relative to baseline. Indicated is the number of channels involved at each time-frequency point. Black box indicates window of interest used for averaging. (A) Cluster showing extent of early theta response. Window from 5–10 Hz, and 200–800 ms. (B) Cluster showing extent of late spindle/beta response. Window from 11–27 Hz, and 700–1200 ms.

For power spectral analysis, we did not observe any time-frequency-channel clusters indicating differential stimulus-related activity during the up and down states. Therefore, we decided to take a more focused approach and directed our attention to two windows of interest where stimulus-evoked activity was greatest. We determined the extent of these windows by first averaging stimulus-evoked responses for the up and down states; that is, we averaged [up-targeted minus fake-up] and [down-targeted minus fake-down]. Then, we looked for clusters showing significant power modulations compared to zero, indicating brain responses to this undifferentiated stimulus category. We observed two significant clusters of stimulus-related power enhancements encompassing the entire scalp: an early theta response and a later spindle/beta response (both P<0.001). We then generated time-frequency heat maps of the number of involved channels in these two significant clusters (Figure 5AB). We visually inspected these plots and averaged power values within two selected windows of interest (theta: 5–10 Hz, 200–800 ms; spindle/beta: 11–27 Hz, 700–1200 ms). While the early theta-window did not reveal differential neural processing for up- and down-targeted stimuli (Figure 4B), there was significantly greater spindle/beta band activity in response to up-targeted stimuli as compared to down-presented sounds in a right frontotemporal area (P<0.001), and additionally, in a smaller left occipital area (P = 0.025) (Figure 4C). We note, however, that t values for this spindle/beta effect were positive across the entire scalp, suggesting that these differences may have been more global than the observed localized clusters indicate. Interestingly, in terms of timing, the spindle/beta response corresponds with the timing of the ERP effect. In summary, when accounting for activity associated with spontaneous SOs, up state-presented stimuli elicit a greater positivity and more spindle/beta activity than down state stimuli. Intriguingly, these effects are rather delayed with respect to stimulus onset, seemingly occurring in the up state after the one in which the stimulus was presented.

### Wake: memory

After allowing participants 30 min to overcome the effects of sleep inertia, they were informed that sounds had been presented during their nap. Then, all 40 sleep-presented, as well as 20 novel sounds were played to the fully awake subjects. Sounds were presented in pairs (one sound at a time), each pair containing stimuli from two different conditions (up/down/novel). Subjects were asked to perform a two-alternative forced choice as to which sound in a pair appeared most familiar to them. Each sound was presented twice, both times paired with a different sound (see Methods - Memory task). Results did not show indications that any category was chosen over any other category consistently (Table 1). We also performed these analyses based on just the first presentation of every sound, in case the first round of presentations had rendered sounds from the different categories more similar in terms of familiarity. However, results were very similar. That is, the SO phase during which sounds were presented during sleep did not appear to drive behavioral responses in any way. Additionally, we checked whether up and down categories combined were chosen more often than novel sounds, suggesting sleep-learning independent of the SO phase. However, this did not appear to be the case either [t(11) = −0.50; P = 0.62]. Subjects also evaluated their confidence in the responses they had provided on a 5-point scale. Ten subjects reported being 'very unsure' or 'unsure' about their choices, one reported being 'sure', and one did not provide an answer. However, scores of the 'sure' responder did not indicate above-chance performance.

**Table 1:** Behavioral performance in a forced choice task.

	up vs. down	up vs. new	down vs. new
percentage [mean ± SD]	48.3±8.3	49.6±10.1	47.9±11.8
statistics [t(11); P]	−0.69; 0.50	−0.14; 0.89	−0.61; 0.55

Indicated is the percentage of choosing the first category over the second. One-sample t tests against 50 (chance level) suggest reported sound familiarity during wakefulness did not depend on whether sounds were novel, or had been presented during SO up or down states of the preceding nap.

**Figure 6:**
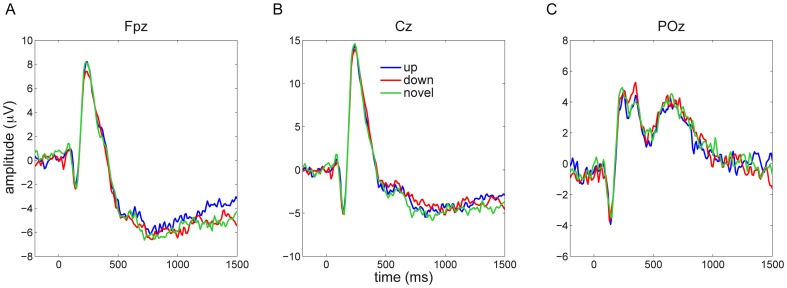
Event-related potentials during wakefulness to novel sounds and to sounds previously presented in SO up and down states. Responses to these three conditions were highly similar across frontal (A), central (B) and posterior (C) channels.

**Figure 7:**
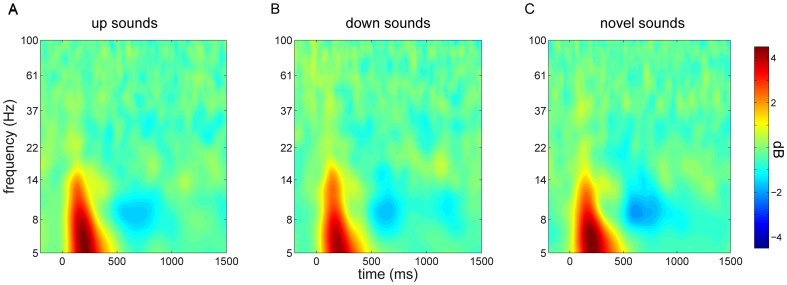
Stimulus-related time-frequency power responses during wakefulness for central channel Cz. Responses to sounds originally presented in the SO up state (A), down state (B), and to sounds not previously played during sleep (C).

During the waking test session EEG was also recorded. As during sleep, we analyzed both ERP and time-frequency power responses across all electrodes for the different stimulus categories (up/down/novel). ERP responses did not provide indications of time-channel clusters of differential neural responses for the different stimulus conditions, neither with an F test across the three conditions, nor with pairwise t tests (Figure 6). Similarly, power responses were highly consistent among conditions (Figure 7), and, again, neither an F test across all conditions, nor pairwise t tests resulted in time-frequency-channel clusters showing reliable differences (closest to significance was a very late [1100–1500 ms] cluster of higher theta power in the up vs. down condition, P = 0.075; all other P>0.5).

**Figure 8:**
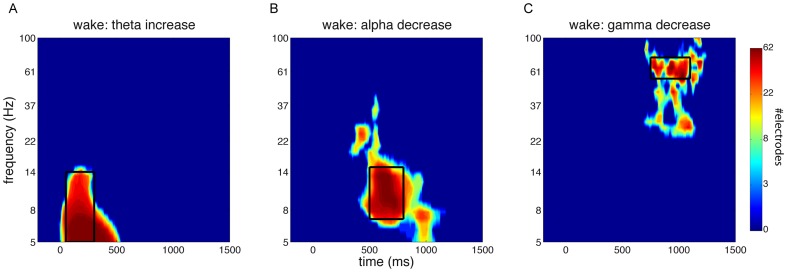
Heat maps of clusters showing significantly modulated power relative to baseline during wakefulness. Indicated is the number of channels involved at each time-frequency point. Black box indicates window of interest used for averaging. (A) Cluster showing extent of early theta increase. Window from 5 - 14 Hz, and 50–300 ms. (B) Cluster showing extent of medium-latency alpha decrease. Window from 7–15 Hz, and 500–800 ms. (C) Cluster showing extent of late gamma decrease. Window from 55–75 Hz, and 750–1100 ms.

We repeated these analyses focusing on windows of interest where there were significant waking stimulus-related power modulations. Similar to our approach during sleep, we determined the extent of these windows by first averaging across all three conditions, assessing clusters showing significant stimulus-related activity, and plotting for every cluster the number of electrodes involved at each time-frequency bin. Stimulus-related power effects were apparent as early theta enhancements (P<0.001), medium-latency alpha decreases (P = 0.004), and late gamma decreases (P = 0.015) across the entire scalp (Figure 8). We designated windows of interest for these theta (5–14 Hz, 50–300 ms), alpha (7–15 Hz, 500–800 ms), and gamma effects (55–75 Hz, 750–1100 ms). However, comparing power values, averaged within each of these windows, among the three conditions did not yield reliable results, again neither using F tests nor pairwise t tests. In other words, there were no indications that prior sound presentation in a specific SO phase during sleep led to altered neural processing during subsequent wake testing.

## Discussion

Using a novel algorithm to deliver sound stimuli to the desired phase of SOs during sleep in real-time, we here demonstrate differential neural processing for auditory information arriving in the SO up and down states. This effect was evident both in time-averaged responses and in time-frequency power modulations. However, subsequent probing during wakefulness with these, as well as novel, stimuli did not reveal neural indices of differential brain responses, nor was behavior affected. Thus, while the SO phase importantly co-determines immediate stimulus processing, this effect does not appear to carry over into wakefulness, suggesting our paradigm did not allow the formation of stable long-term memories during deep sleep.

First, our findings show that both up- and down-targeted stimulus presentation result in evoked potentials distinct from naturally occurring SOs. More specifically, stimuli in both conditions elicited an early (∼300 ms) positive response (Figure 4A), which, in terms of timing and amplitude, is in line with auditory evoked potentials during wakefulness (see also Figure 6). Furthermore, in both conditions, this early response was followed by a sharp, high-amplitude, down state-like deflection around 600 ms, and an ensuing up state-like positivity. Relative to spontaneous up states, up state-presented sounds evoked, first, an enhanced up state, second, a more rapid and sharper down state, followed by, third, an enhanced late positivity (Figure 2B). Compared with naturally occurring down states, down state-delivered stimuli led to, first, a small positive-going deflection, followed by, second, a delayed, and more pronounced, down state (Figure 2C). Notably, these ERP patterns in both the up- and down-targeted conditions are consistent with a large literature on stimulus-evoked K-complexes, discussed in more detail below [12], [31].

Power responses to stimuli directed at both up and down states encompassed an early theta response, maximal around 500 ms, followed by a delayed spindle/beta response around 1000 ms post-stimulus (Figure 3BC). The first effect is centered right before the trough of the ERP down state-like deflection, while the timing of the latter effect appears to correspond to the late ERP positivity, which is in line with the grouping influence of up states on faster brain activity [17], [19].

Comparing up- and down-targeted stimuli directly, our data show that, when correcting for spontaneous SOs, up-targeted stimuli lead to a large and sustained positivity from 500 ms onwards (Figure 4A). Timing-wise, this effect matched with robustly enhanced spindle/beta activity for up-targeted stimuli (Figure 4C), relative to down-targeted ones. Combined, these findings suggest sound delivery in the up state elicits a second up state that occurs sooner in time, has higher amplitude, and features more prominent fast brain oscillations, than with stimulus presentation in the down state.

It is interesting to compare our findings to another study relating sound-evoked brain responses to the SO phase [16]. In that study, simple tones presented in a 300 ms window after the peak negativity led to a greater evoked positivity around 200 – 500 ms post-stimulus than tones presented in the 300 ms before the down state. This ERP effect occurred alongside increased hemodynamic activity in secondary auditory cortex, possibly suggesting enhanced stimulus processing for up state-presented stimuli. In our data, we observed ERP responses with similar early peaks around 300 ms, but these were not different for up- and down-targeted stimuli (Figure 4AC). Rather, we observed a much later difference (500–1200 ms) seemingly related to the succeeding up state, constituting a different effect altogether. These discrepancies may be due to many factors, such as differences in stimulus material, analysis methods, and scanner noise. One other study investigated somatosensory evoked potentials as a function of SO phase [15]. However, all reported effects are on evoked components occurring <150 ms after stimulus onset, making it problematic to compare these authors' findings and ours sensibly.

Comprehending the functional relevance of our findings is challenging. First, it appears both up- and down-targeted stimuli elicit K-complexes, in particular the late, positive aspect thereof, along with associated spindle and beta activity. In addition, this effect appears more vigorous for up-targeted stimuli. The physiological importance of K-complexes is still a matter of contention. Some have suggested that K-complexes impose periodic excitatory and inhibitory effects on cortical and thalamic neurons, essentially equating K-complexes with SOs [32]. Indeed, it has been shown that SO down states and K-complex troughs are highly similar in terms of their cortical generators [33]. Others suggest the dependence of K-complex morphology on sensory stimulation characteristics is more in line with a sleep-protective role [34], a notion supported by findings relating K-complexes with hemodynamic cortical deactivations [35]. In a similar vein, functionally distinct roles have been proposed for thalamocortical spindles. On the one hand, spindles have been thought to suppress the transmission of auditory information to cortex, as suggested by altered event-related responses [13], [14] and reduced hemodynamic activity [16], [36] following sound presentation in the presence of spindles. On the other hand, strong links between spindles and memory performance have been described [37], [38]. While some of these ties might be explained in terms of trait correlations between spindle characteristics and learning ability [39], other findings clearly link spindle responses to specific memory reactivations [9] and memory retention [38], implicating spindles in processes of memory consolidation.

Taken together, several interpretations of our findings are possible. First, more pronounced K-complexes and spindle activity following up state stimulus presentation may reflect the sleep-protective function of both these signature brain waves. Perhaps the need to suppress sensory processing and prevent waking is stronger in the up state, when neuronal networks are relatively depolarized. Second, given the aforementioned relevance of sleep spindles in memory consolidation, enhanced spindle and beta activity following up state sound stimulation may reflect heightened immediate reprocessing of that stimulus. In this respect it is noteworthy that the stimulus-evoked increase in spindle activity appeared not in the up state when sound playback occurred, but in the next up state. Thus, stimulus reprocessing, in terms of spindle and beta activity, may have been postponed until the next up state permitted it. Finally, the algorithm's phase predictions were better for up than for down states. Thus, the possibility should be entertained that greater phase variability in the down-targeted condition may have led to greater variability in responses, less-defined average evoked responses, and, consequently, reduced activity compared to the up-targeted condition. We believe this to be unlikely, however, since down-targeted stimuli also elicited a more negative peak than up-targeted sounds around 600 ms, a finding clearly arguing against the idea that phase variability resulted in dampened responses.

Considering the post-sleep testing session in more detail, we did not observe indications that stimuli were processed differently depending on whether they had been presented during sleep or were entirely new, or depending on whether they had been presented in the SO up or down state. This lack of an effect was apparent for both behavioral performance and EEG-based measures. For behavioral testing, we made use of a two-alternative forced choice test. Importantly, this paradigm does not require subjects to consciously remember previous encounters with the stimulus material. Rather, because participants are forced to make a decision, any kind of memory, regardless of its nature, can potentially bias behavior. Thus, it is generally viewed as a very sensitive way of determining whether information (i.e., memories) is present [40].

Regarding EEG measures during wakefulness, these did not reveal any differential neural processing either. In the absence of behavioral evidence of memory, a neural effect could still have been construed as a sign of sleep-learning because of the persistence of differential stimulus processing from sleep into wakefulness. For example, the right hemisphere has been shown to be able to track the presentation history of sound objects over a 2 h period [41]. This effect was manifested as repetition suppression of the auditory evoked response, in which repeated encounters with a stimulus elicit progressively weaker responses. Similar effects have been observed in the visual domain as reduced gamma oscillatory activity to repeated stimuli [42]. These phenomena are thought to rely on the gradual 'sharpening' of neural stimulus representations [43]. Such repetition mechanisms might have been expected to be influenced by the SO phase, resulting in residual differential neural responses during wakefulness. However, neither ERPs nor time-frequency power responses distinguished among stimulus categories during the waking test.

A few recent studies reported it is possible to establish new memories during sleep. One report demonstrated that sounds could be conditioned to odors of positive and negative valence, as assessed by discriminant sniff responses lasting into wakefulness [6]. Another one showed fear extinction learning during sleep is possible [7]. A major difference between these studies and ours regards the neural structures likely to be involved. Whereas classical conditioning and fear extinction rely heavily on deep-lying regions (hippocampus, amygdala, brain stem), any behavioral effect in our design would likely have to arise through neocortical involvement. That is, complex auditory representations, such as the ones engendered by our stimuli, are known to have a cortical basis [44]. Consequently, both behavioral and EEG-based effects during wake testing would largely have to rely on memory formation in neocortical circuits. Indeed, our entire approach was aimed at investigating the role of SOs in the processing and encoding of neocortically based information, since one would not expect the phase of neocortical SOs to be very relevant for learning-related processes taking place at the subcortical level.

For these reasons, one suggestion is that while it may be possible to acquire new information during sleep, the type of memories that can be formed are limited to those that do not rely on neocortical networks. This may be due to the vastly different cortical neuromodulatory levels during sleep, in particular during SWS, when the availability of certain neurotransmitters and hormones is markedly reduced. Both low levels of acetylcholine [45] and cortisol [46] are known to affect encoding adversely, possibly rendering SWS a state incompatible with neocortical memory formation. Another reason may be that while up- and down-targeted stimuli were neurally differentiated during sleep, individual sounds were not. That is, brain responses during sleep to both stimulus categories may have been different, but nonspecific, without individual sounds being processed by the corresponding semantic networks. Consequently, memories for individual stimuli would also not have been formed. Alternatively, learning of neocortically based information might be possible in principle, but we may simply not have presented sounds sufficiently often to leave a permanent mark on neural circuitry. Thus, implementing a longer sleep period, with more SOs, would allow many more repetitions of individual stimuli. On the other hand, with three sleep-presentations per sound on average, one would expect the brain to be able to express signs of repetition suppression. As a final possibility, it may be that our null-results during wakefulness reflect waking interference effects unrelated to sleep. That is, in the interval between waking up and testing, participants experienced regular auditory input, potentially neutralizing effects that were present immediately following waking. However, such interference would have to be very general, since, prior to the memory task, subjects did not encounter stimuli in our quiet laboratory environment similar to the ones played during sleep.

Regarding the technical aspects of this study, we employed a sophisticated, novel algorithm able to predict any desired phase of the SO. The algorithm we developed was quite successful at phase prediction, although up states were predicted more accurately than down states. While we do not know the definitive cause, it may be surmised that the difference in morphology between up and down states is a factor. Specifically, down states are generally ‘sharper’ than the more gradual, ‘rounder’ up states. As a result, the algorithm's sine fitting procedure may be less ideal for down state prediction, and alternative fitting approaches may be considered. Nonetheless, we observed highly distinct phase distributions for up- and down-targeted stimuli, attesting to the algorithm's validity. Naturally, the algorithm can be employed in numerous experimental designs where stimulus delivery is desired to occur consistently in a specific phase of a selected frequency band. For SOs, this could involve other approaches for investigating sleep-learning, or protocols for reactivating previously encoded memory traces [47]. While in the current study we used a prefrontal electrode to base our phase predictions on, it is possible to use SOs from any electrode deemed theoretically relevant for phase prediction, permitting the pursuit of a multitude of research questions.

Indeed, in the present study, an alternative approach would have been to base phase predictions on an electrode site over auditory cortex, which may have been more sensitive a region for SO phase-dependent sound processing and retention. However, given the ongoing development of our algorithm, we opted for the brain area with the most pronounced SOs (i.e., frontal cortex [48]). Another complexity is that individual SOs are known to have a unique traveling profile [48]–[50], implying that SO phase relations between pairs of electrodes vary across SO cycles. Thus, SO propagation trajectories likely impacted our results in that up- and down-targeted stimuli may have had a consistent relation with the phase of frontal SOs, but probably a more variable one with SOs over cortical areas further away. However, the fact that most effects encompassed the majority of electrodes, including scalp regions far removed from frontal cortex, suggests we capitalized on rather global SOs.

In conclusion, we show that the phase of the slow oscillation in deep sleep has a decisive influence on immediate stimulus processing. However, differential neural processing did not carry over into wakefulness or affect behavioral decisions, suggesting the possibility for sleep-learning is limited.
